# Changes in rhizosphere microbial community of potato under farmland with different cultivation years in alpine-cold regions

**DOI:** 10.7717/peerj.21205

**Published:** 2026-07-06

**Authors:** Xudong Lu, Zhongren Yang, Fenglan Zhang, Xiaoyan Zhang, Dong Zhang, Xiumei Huang, Chun Yin

**Affiliations:** 1 Vocational and Technical College, Inner Mongolia Agricultural University, Baotou, Inner Mongolia, China; 2 College of Horticulture and Plant Protection, Inner Mongolia Agricultural University, Hohhot, Inner Mongolia, China; 3 Inner Mongolia Academy of Agricultural and Animal Husbandry Sciences, Hohhot, Inner Mongolia, China

**Keywords:** Alpine-cold region, Cultivation years, Edaphic factors, Microbial community, Microbial functions, Rhizosphere

## Abstract

**Aim:**

Soil quality and microbial community structure are highly responsive to tillage practices, with long-term tillage leading to soil degradation and shifts in the rhizosphere microbial community. However, the mechanisms through which tillage duration and soil quality influence the potato rhizosphere microbial community in the black soil regions of alpine areas remain poorly understood.

**Methods:**

To address this, farmlands with varying tillage durations (0, 5, 20, and 60 years) were selected. The space-for-time substitution method was employed to investigate the dynamic variations and functions of the potato rhizosphere microbial community across these different tillage durations, aiming to elucidate microbial succession patterns under prolonged conventional tillage.

**Results:**

The results revealed that long-term tillage significantly altered soil nutrient content. The Chao and Shannon indices for bacteria varied in response to changes in soil nutrients, whereas those for fungi declined with increasing tillage duration and eventually stabilized. Soil alkaline nitrogen (AN), total nitrogen (TN), total phosphorus (TP), and organic matter (SOM) content were positively correlated with the beneficial bacterial community. Distinct differences in bacterial biomarker taxa were observed across tillage durations, while the number of fungal biomarker taxa sharply decreased with extended tillage. Long-term tillage notably increased the abundance of aerobic chemoheterotrophic bacteria, ureolytic bacteria, organic matter-decomposing bacteria, fungal plant pathogens, and endophytic fungi. Partial least-squares analysis indicated that tillage duration and soil factors collectively explained 72% of bacterial diversity and 54% of its compositional variation, as well as 54% of fungal diversity and 72% of its compositional variation. Both factors exerted direct and indirect regulatory effects on the microbial community.

**Conclusion:**

In summary, long-term tillage had a direct impact on the rhizosphere microbial community and its functional potential, with bacteria being more sensitive to long-term tillage than fungi. Thus, in the development of the potato industry in the black soil regions of alpine areas, maintaining and improving soil fertility and quality should be prioritized.

## Introduction

Tillage, a common farmland management practice, artificially disturbs the soil to optimize crop growth and enhance production. It plays a beneficial role in weed control, improving nutrient utilization, and increasing the incorporation of crop residues into the soil (Das et al., 2014). However, the most significant negative impact of long-term traditional tillage on soil is the degradation of soil quality. Specifically, soil structure (Hernández et al., 2019; Sun et al., 2016; Vizioli et al., 2021) and nutrient characteristics (Sokolowski et al., 2020; Tshuma et al., 2021) deteriorate as tillage duration increases (González-Chávez et al., 2010; Puerta et al., 2019). Prolonged tillage not only affects soil quality but also induces shifts in soil microbial communities. Soil microorganisms, vital components of agricultural ecosystems, regulate nutrient cycling, contribute to soil structure formation, and support crop growth (Helliwell et al., 2014; Nimmo, Lynch & Owen, 2013). A diverse microbial community is essential for maintaining soil ecosystem stability (Waldrop et al., 2017), but these microorganisms are highly sensitive to agricultural practices, particularly the soil environmental changes induced by tillage. Fungi play a pivotal role in soil quality by promoting aggregation and decomposing plant residues. There is a strong correlation between the intensity of soil disturbance from long-term traditional tillage and the decline in fungal Phospholipid-derived fatty acid (PLFA) biomarkers, particularly arbuscular mycorrhizae (Balla Kovács et al., 2024; Wang et al., 2012). In sandy soils, traditional tillage may increase the abundance of Proteobacteria and Planctomycetes compared to no-tillage, significantly impacting the abundance of nitrifiers and denitrifiers (Hao et al., 2019; Zhang et al., 2022). In black soils, long-term tillage reduces the relative abundance of nitrifying and nitrogen-fixing bacteria while increasing the abundance of carbon-degrading bacteria (Liu et al., 2022). Under traditional tillage, bacteria dominate crop residue decomposition, and soil disturbance may favor aerobic microorganisms (Wang et al., 2012), such as an increased abundance of actinomycetes (Ramirez-Villanueva et al., 2015). Additionally, traditional tillage leads to a decrease in soil microbial biomass and shifts the microbial community structure, particularly in surface soils (Minoshima et al., 2007). Long-term studies have shown that traditional tillage can reduce populations of Pseudomonas and Agrobacterium, impairing biological nitrogen fixation in soil (Wang et al., 2020). Tillage practices significantly alter the microbial community structure (Li et al., 2021), affecting both the rhizosphere and non-rhizosphere regions. The rhizosphere is a specialized zone where crops and microorganisms interact (Mendes, Garbeva & Raaijmakers, 2013). Initially, the diversity of the rhizosphere microbial community is similar to that of the non-rhizosphere soil community. However, as the crop life cycle progresses, activities such as planting and growth recruit microorganisms from the surrounding soil into the rhizosphere, forming a crop-associated microbial community (Chaparro, Badri & Vivanco, 2014; Edwards et al., 2018; Yan et al., 2020b; Zimudzi et al., 2018). This rhizosphere process, particularly the crop-associated microbial community (Mariotte et al., 2018), is significantly influenced by artificial tillage practices. Thus, understanding the effects of tillage on soil microorganisms is critical for sustainable farmland management. While current research primarily focuses on the relationships between tillage systems, tillage methods, crop types, soil properties, and microorganisms (Li et al., 2021; Liu et al., 2022; Qin et al., 2022; Sun et al., 2023), studies examining the impact of long-term tillage on crop rhizosphere microorganisms remain limited.

Potato (*Solanum tuberosum* L.) has become the fourth most important food crop globally, following rice, maize, and wheat (Li et al., 2022; Pfeiffer et al., 2017). Current research on the relationship between potato and microorganisms has mainly focused on correlations under different planting patterns (Gu et al., 2022; Ma et al., 2015; Qin et al., 2022), tillage systems (Djaman et al., 2022), cultivars (Gu et al., 2022; Pfeiffer et al., 2017), and soil properties (Tian et al., 2023). However, studies on the effects of different tillage durations on the rhizosphere microorganisms of potato remain scarce.

The western foothills of the Greater Khingan Mountains, located in a cold region, are a major area for black soil cultivation in Northeast China. The region experiences an average annual temperature ranging from −0.1 °C to 4.0 °C, with a frost-free period of 70–95 days and annual precipitation of 250–300 mm, supporting one crop per year. These conditions make it ideal for potato seed production, establishing it as an important commercial potato production base in China (Li et al., 2024). However, long-term conventional tillage has led to soil degradation, impairing the stability of the farmland soil microbial community. Therefore, understanding the response mechanisms of potato rhizosphere microorganisms to soils with different tillage durations is essential for the healthy and sustainable development of local agriculture. On this basis, the present study puts forward the following research hypothesis: With the extension of cultivation years, changes in soil quality in black soil farmlands of alpine cold regions will significantly drive regular variations in the community composition, diversity, and functional characteristics of potato rhizosphere microorganisms, which exhibit a specific correlation pattern with cultivation duration. The research objectives are as follows: (1) To clarify the differential characteristics of soil physicochemical properties under different cultivation years; (2) To reveal the succession patterns of rhizosphere microbial community structure, diversity, and functional genes during potato cultivation in farmlands with different cultivation histories; (3) To elucidate the coupling mechanism among cultivation years, soil quality, and key taxa of rhizosphere microbial communities, so as to provide a theoretical basis for sustainable cultivation and soil quality improvement of potato farmlands in alpine black soil regions. To this end, the study investigated the mechanisms of changes in the potato rhizosphere microbial community in farmlands with varying tillage durations in cold regions using a “space-for-time” approach.

## Materials & Methods

### Rhizosphere soil sample collection

The sampling site is located at Yunmaixing Farm, Mianduhe Town, Yakeshi City, Inner Mongolia Autonomous Region (49°11′25.4017″–49°12′32.5938″N, 120°57′29.2040″–120°58′31.0880″E).

This area is characterized by a boreal cold temperate continental monsoon climate, with an average annual temperature of approximately −2.1 °C and annual precipitation ranging from 250–300 mm. The predominant soil type is chernozem. The test crop is potato, specifically the cultivar Favorita.

**Figure 1:**
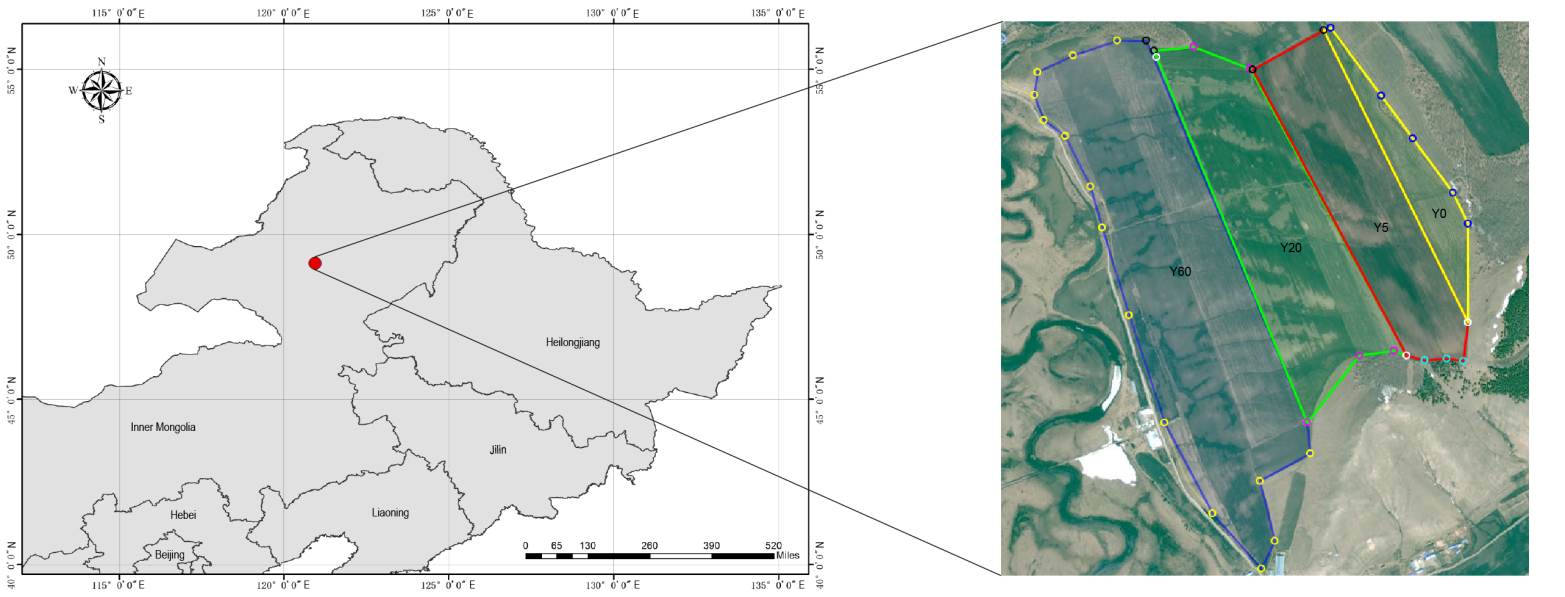
Experimental site.

Sampling sites were established in farmlands with varying cultivation durations (0, 5, 20, and 60 years) within the farm. Over the past 60 years, the crops planted and field management practices have remained consistent. The 0-year plot represents newly reclaimed natural land, and the layout of farmlands with different cultivation durations is shown in Fig. 1. Uniform field management practices were applied to all potato plants. Potatoes were sown in early May 2022, with a plant spacing of 25 cm and a row spacing of 90 cm. The fertilization rates were as follows: nitrogen fertilizer (N) at 300 kg/hm^2^, phosphorus fertilizer (P_2_O_5_) at 150 kg/hm^2^, and potassium fertilizer (K_2_O) at 150 kg/hm^2^. Samples were collected in October 2022 during the same growth stage of the potatoes. The five-point sampling method was employed at each farmland plot. Rhizosphere soil samples from five potato plants were collected at each sampling point. After mixing the samples from each treatment, three replicates were set (*n* = 3), yielding a total of 12 soil samples from the four treatments.Whole potato plants, along with the attached soil, were carefully excavated using a clean shovel, and soil from the 0–20 cm depth around the potato roots was collected. After removing visible residues, the soil was used for measuring physical and chemical indices, with the samples stored at −20 °C for physicochemical analysis. Rhizosphere soil, tightly adhering to the potato roots within one mm, was collected using a sterile brush. The samples were uniformly mixed, placed in 50 mL centrifuge tubes, and transported to the laboratory on dry ice. After removing visible residues, the samples were stored at −80 °C for microbial analysis.

### Soil physicochemical analysis

Soil organic matter (SOM) was determined using the potassium dichromate volumetric method combined with external heating; total nitrogen (TN) was measured using the Kjeldahl method; total phosphorus (TP) and potassium were determined through digestion with sulfuric and perchloric acids; alkali-hydrolyzable nitrogen was measured using the alkali hydrolysis diffusion method; available phosphorus (AP) was extracted with 50 mL NaHCO_3_ and determined by spectrophotometric colorimetry; and available potassium (AK) was extracted with ammonium acetate and measured by flame photometry. All soil indices were determined according to the methods outlined in Soil and Agricultural Chemistry Analysis (Bao, 2000).

### DNA extraction, library construction, and metagenomic sequencing

DNA extraction from the samples was performed using the Mag-Bind^®^ Soil DNA Kit (Omega Bio-Tek, USA). The integrity of the extracted genomic DNA was assessed *via* 1% agarose gel electrophoresis. DNA fragmentation was carried out using the Covaris M220 (Nanjing Genesky Biotechnologies, China), selecting fragments approximately 300 bp in length. A DNA library was constructed using the TruSeq™ DNA Sample Prep Kit, following these steps: ligation of “Y”-shaped adapters, removal of adapter self-ligated fragments through magnetic bead screening, enrichment of library templates by PCR amplification, denaturation with sodium hydroxide to generate single-stranded DNA fragments, amplification by PCR, and sequencing using the Illumina Novaseq platform.

For quality control, Fastp (Chen et al., 2018) (https://github.com/OpenGene/fastp, version 0.20.0) was used to trim adapter sequences from both the 3′ and 5′ ends of the reads. Reads shorter than 50 bp post-trimming, with an average base quality value below 20, or containing N bases, were discarded. High-quality paired-end and single-end reads were retained. BWA (Li & Durbin, 2009) (http://bio-bwa.sourceforge.net/, version 0.7.9a) was used to align the reads to the host DNA sequence, and contaminant reads with high alignment similarity were removed. Sequence assembly was performed using Megahit (https://github.com/voutcn/megahit) at different sequencing depths, with contigs ≥ 300 bp selected as the final assembly output. Prodigal (Hyatt et al., 2010) (https://github.com/hyattpd/Prodigal) was employed to predict open reading frames (ORFs) from the assembled contigs. Genes ≥ 100 bp were selected and translated into amino acid sequences.

To cluster the predicted gene sequences from all samples, CD-HIT (Fu et al., 2012) (https://cd-hit.org (accessed June 12, 2026), version 4.6.1) was used with parameters set at 90% identity and 90% coverage. The longest gene in each cluster was taken as the representative sequence to construct a non-redundant gene set. The high-quality reads from each sample were aligned with this non-redundant gene set (95% identity) using SOAPaligner (Li et al., 2008) (https://anaconda.org/bioconda/soapaligner/files, version 2.21), and the abundance of genes in each sample was calculated.

The amino acid sequences of the non-redundant gene set were aligned with the NR database using Diamond (Buchfink, Xie & Huson, 2015) (http://www.diamondsearch.org/index.php, version 0.8.35) *via* BLASTP with an e-value threshold of 1e−5. Taxonomic annotation was derived from the NR database, and species abundance was calculated based on the sum of gene abundances corresponding to each species.

### Data analysis

The Student’s *t*-test was applied to assess microbial α-diversity, including the Shannon and Chao indices, across different tillage durations. Non-metric multidimensional scaling (NMDS), based on Bray-Curtis dissimilarity matrices, was used to evaluate microbial β-diversity. Linear discriminant analysis effect size (LEfSe) identified taxa with significantly different abundances across tillage durations. The non-parametric Kruskal-Wallis sum-rank test was first used to detect features with significant abundance differences, followed by the Wilcoxon rank-sum test to confirm the consistency of differentially abundant taxa. Linear discriminant analysis (LDA) was then applied to estimate the effect size of these taxa in distinguishing groups, with significance set at *p* < 0.05 and an LDA score > 4.0. Redundancy analysis (RDA) was conducted to examine the relationship between microbial distribution and environmental factors. Mantel test correlation analysis between environmental variables and microbial communities was performed using the vegan package (version 2.4.3) in R 3.3.1. Spearman’s correlation analysis was employed to determine the correlation coefficients between rhizosphere microorganisms and soil factors. The partial least squares method was used to analyze and evaluate the effects of cultivation duration and soil factors on fungal and bacterial communities. A goodness-of-fit (GoF) value greater than 0.36 was considered to indicate a good model fit. Path coefficients and the coefficient of determination (R^2^) of the path model were calculated using the PLS package in R 4.4.2. FAPROTAX (Louca, Parfrey & Doebeli, 2016) and FUNGuild (Nguyen et al., 2016) were used to predict the potential functional traits of bacteria and fungi, respectively.

## Results

### Changes in soil parameters of farmland under different tillage years

As cultivation duration increased, TN, TP, organic matter, and available nitrogen (AN) contents in the Y0 and Y5 plots were significantly lower than those in the Y20 plot (*p* < 0.05) (Table 1). Among these, TN, TP, organic matter, and AN were lowest in Y5 and highest in Y20, with values ranging from 1.46 to 2.42 g/kg, 0.47 to 0.64 g/kg, 33.11 to 53.9 g/kg, and 240.8 to 244.3 mg/kg, respectively. Total potassium content was lowest in Y5 and highest in Y60, ranging from 8.42 to 10.32 g/kg. AP content was highest in Y0 and lowest in Y5, ranging from 1.16 to 9.48 mg/kg, while AK content was highest in Y5 and lowest in Y60, ranging from 290 to 385.83 mg/kg.

**Table 1:** Soil sample information of farmland with different cultivation years.

NO	Sampling location	Cultivation age	TN (g/kg^−1^)	TP (g/kg^−1^)	TK (g/kg^−1^)	SOM (g/kg^−1^)	AN (mg/kg^−1^)	AP (mg/kg^−1^)	AK (mg/kg^−1^)
Y0	120°58′31.5526″E,49°12′11.9276″N	0	2.04 ± 0.09bc	0.56 ± 0.02b	9.48 ± 0.01a	52.49 ± 0.35a	240.8 ± 15.4ab	9.48 ± 0.04 a	330 ± 18.3 b
Y5	120°58′18.3658″E,49°12′12.1962″N	5	1.46 ± 0.01d	0.47 ± 0.01c	8.42 ± 0.01ab	33.11 ± 3.28c	165.2 ± 18.2c	1.16 ± 0.03 d	385.83 ± 2.5a
Y20	120°58′01.9234″E,49°12′09.1268″N	20	2.42 ± 0.03a	0.64 ± 0.00a	8.67 ± 1.3 a	53.90 ± 1.76a	244.3 ± 4.9 a	2.34 ± 0.15 c	341.67 ± 11.6b
Y60	120°57′40.6772″E,49°12′04.1305″N	60	1.97 ± 0.00c	0.61 ± 0.02a	10.32 ± 0.11a	47.20 ± 0.7 b	233.8 ± 16.8ab	4.95 ± 0.03 b	290 ± 10c

**Notes.**

Y0, Y5, Y20, and Y60 represent soils tilled for 0,5,20, and 60 years. Values represent mean ± standard deviation based three replicates

Different lowercase letters indicate significant difference in different sampling location, *P* < 0.05.

### Diversity and abundance of bacterial and fungal communities under different cultivation years

**Figure 2:**
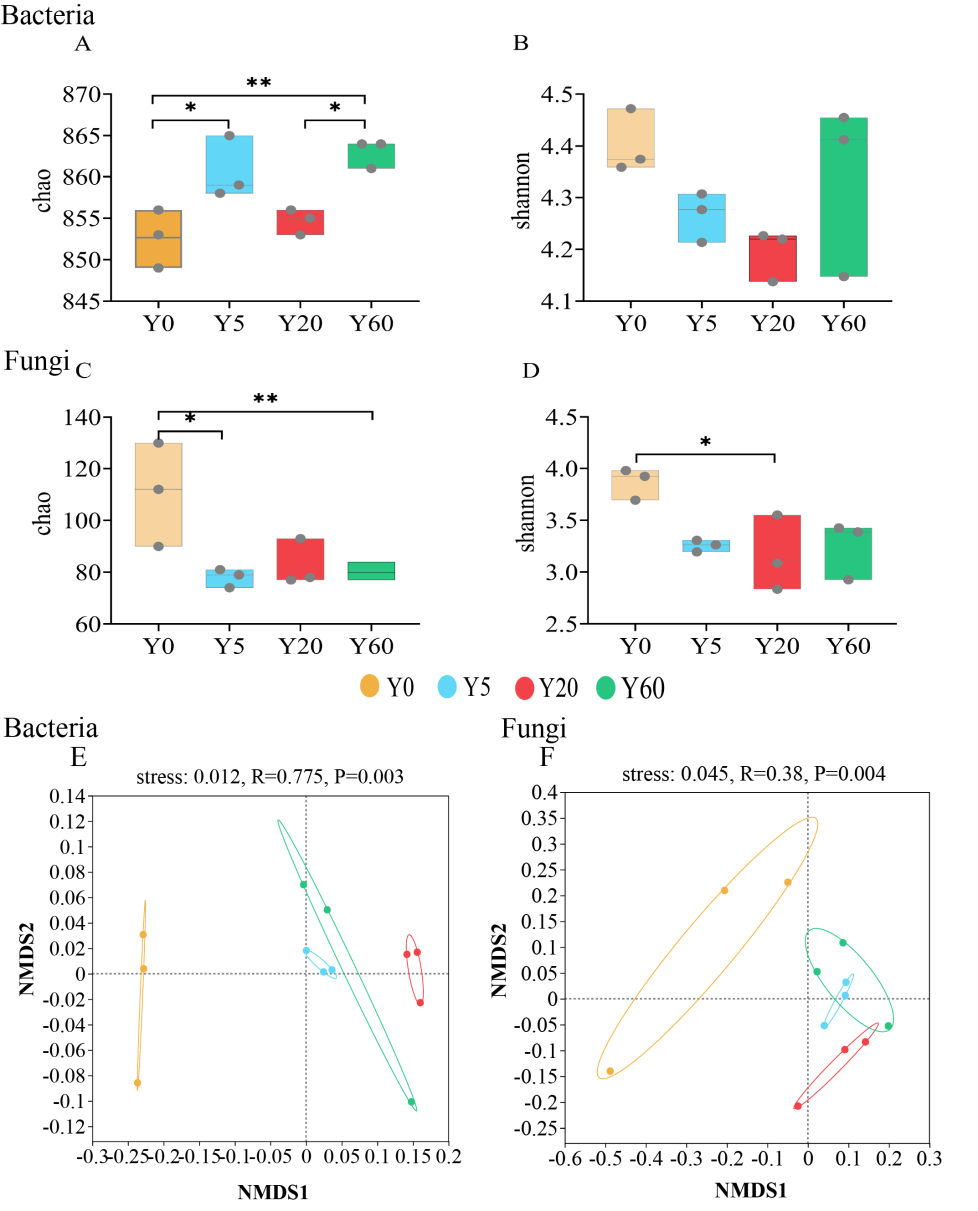
Alpha diversity (Shannon and Chao indices) of rhizospheric bacteria (A, B) and fungi (C, D) under different tillage years. Significant differences among Y0, Y5, Y20, and Y60 sites are shown in each panel (*p < 0.05, **p < 0.01). Non-metric Multidimensional Scaling (NMDS) analysis based on Bray-Curtis distance (E, F) of bacteria and fungi in rhizosphere microbial communities under different tillage durations.

To assess the α-diversity of rhizosphere microorganisms across different tillage durations, the Chao and Shannon indices were calculated. The bacterial Chao index was significantly higher in the Y60 group compared to Y0 and Y20, with Y5 also showing significantly higher values than Y0. No significant differences were observed between Y20 and Y0/Y5, nor between Y60 and Y5. The Shannon index showed no significant differences among the groups (Figs. 2A, 2B). For fungi, the Chao index was highest in Y0, significantly differing from both Y5 and Y60, while the Shannon index peaked in Y0, significantly differing from Y20 (Figs. 2C, 2D). NMDS, based on Bray-Curtis dissimilarity, was performed to visualize the β-diversity of bacterial and fungal communities. Soil bacterial communities under different tillage durations clearly clustered into four distinct groups. ANOSIM tests revealed that tillage duration significantly influenced the composition of bacterial (*R* = 0.775, *p* = 0.003) and fungal (*R* = 0.38, *p* = 0.004) communities (Figs. 2E, 2F), with a marked separation between the Y0 group and the other groups.

### Differences in bacterial and fungal community structures under different cultivation years

**Figure 3:**
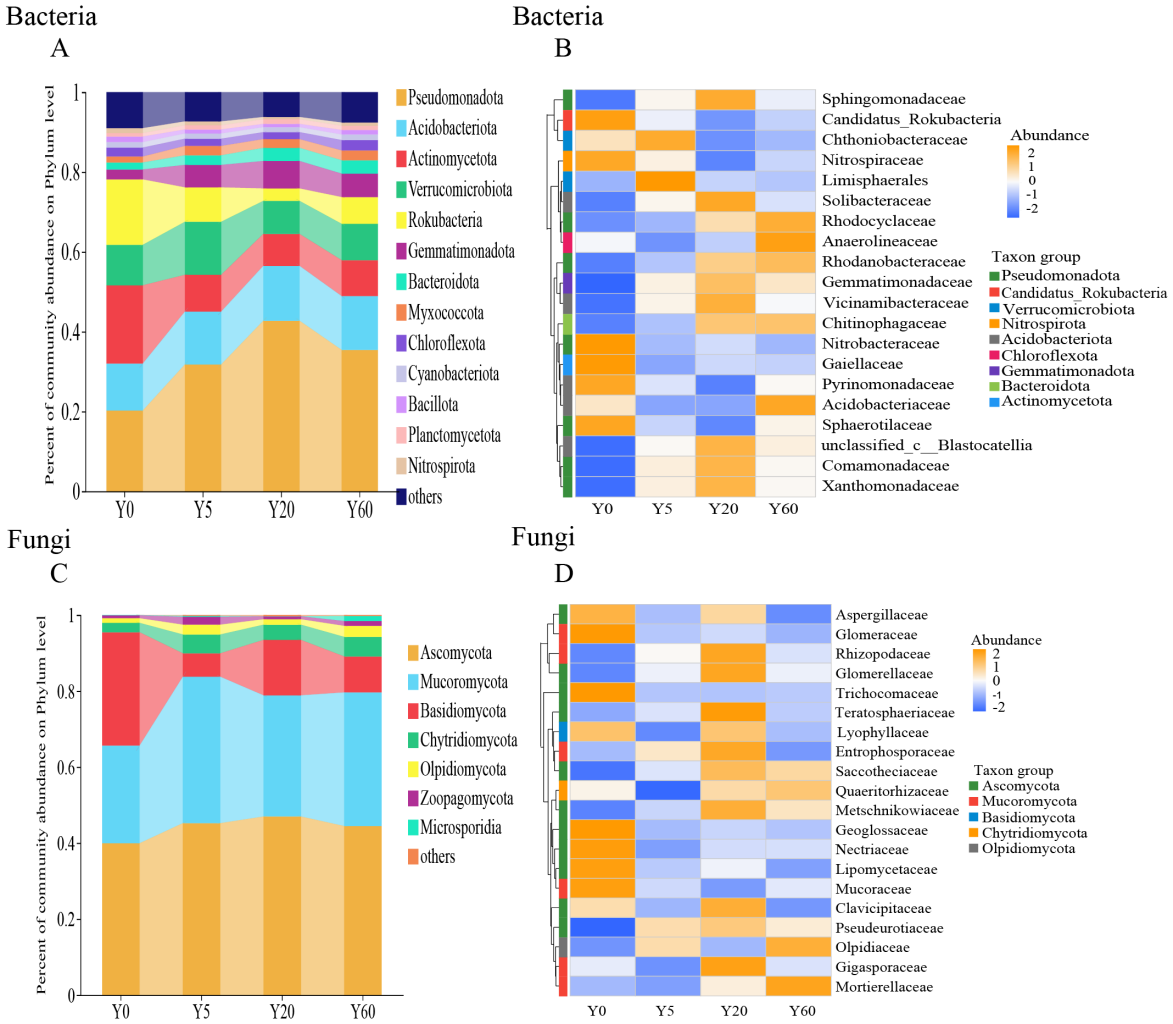
Relative abundances of soil bacteria ((A) phylum level; B: family level) and fungi (A: phylum level; (B) family level) across different cultivation durations. For this analysis, the top 10 phyla and top 20 genera of bacteria and fungi, ranked by relative abundance, were selected respectively.

The impact of different tillage years on microbial taxon composition was examined by analyzing the abundance differences of rhizosphere microbial communities at the phylum level. Bacterial communities across the four tillage years were primarily dominated by *Pseudomonadota* (20.23%, 31.77%, 42.71%, 35.41%), *Acidobacteriota* (11.77%, 13.24%, 13.74%, 13.47%), *Actinomycetota* (19.58%, 9.2%, 8%, 9%), *Verrucomicrobiota* (10.1%, 13.28%, 8.28%, 9.1%), *Candidatus Rokubacteria* (16.38%, 8.62%, 3.11%, 6.69%), *Gemmatimonadota* (2.48%, 5.61%, 6.86%, 5.86%), and *Bacteroidota* (1.74%, 2.41%, 3.29%, 3.35%). Additionally, phyla with relative abundances greater than 1% in the tested soils included *Myxococcota*, *Chloroflexota*, *Cyanobacteriota*, *Bacillota*, *Planctomycetota*, and *Nitrospirota*, while those with <1% abundance accounted for 9%, 7.3%, 6.2%, and 7.6% across groups (Fig. 3A). As tillage duration increased, the relative abundances of *Pseudomonadota*, *Acidobacteriota*, *Verrucomicrobiota*, and *Gemmatimonadota* initially increased before decreasing. Conversely, *Actinomycetota* and *Candidatus Rokubacteria* followed an opposite trend (first decreasing, then increasing). Significant differences in the relative abundances of *Pseudomonadota*, *Gemmatimonadota*, and *Bacteroidota* were observed between Y20 and both Y0/Y5, but not between Y60 and Y5. In contrast, *Acidobacteriota*, *Verrucomicrobiota*, and *Candidatus Rokubacteria* exhibited significant differences between Y0 and Y20/Y60, but not between Y5 (Table S1).

This study analyzed the responses of the top 20 bacterial families (ranked by relative abundance) to cultivation duration. Analysis of variance (ANOVA) revealed that 17 of these families exhibited significant differences across soils with varying cultivation durations (Table S4). The relative abundances of *Sphingomonadaceae*, *Gemmatimonadaceae*, *Vicinamibacteraceae*, *Blastocatellia*, *Comamonadaceae*, *Xanthomonadaceae*, *Chitinophagaceae*, *Rhodocyclaceae*, *Anaerolineaceae*, and *Solibacteraceae* in the Y0 plot were significantly lower than those in the other plots with longer cultivation durations (Fig. 3B). Among the Y5, Y20, and Y60 plots, the relative abundances of these families were significantly higher in the Y20 plot compared to the Y5 and Y60 plots. In contrast, the relative abundances of *Chthoniobacteraceae*, *Candidatus Rokubacteria*, *Sphaerotilaceae*, and *Limisphaerales* were significantly lower in the Y20 plot than in the other three plots.

Fungal communities were primarily composed of Ascomycota (39.92%, 45.21%, 46.96%, 44.45%), *Mucoromycota* (25.71%, 38.53%, 31.88%, 35.17%), *Basidiomycota* (29.81%, 6.15%, 14.58%, 9.44%), *Chytridiomycota* (2.47%, 4.95%, 3.96%, 5.15%), *Olpidiomycota* (1.22%, 2.59%, 1.43%, 2.9%), *Zoopagomycota* (0.78%, 2.05%, 0.77%, 1.25%), and *Microsporidia* (0.03%, 0.24%, 0.02%, 1.16%) (Fig. 3C). The relative abundance of Ascomycota initially increased and then decreased with tillage years, while *Mucoromycota*, *Chytridiomycota*, *Olpidiomycota*, *Zoopagomycota*, and Microsporidia followed a trend of increase-decrease-increase. Basidiomycota displayed a decrease-increase-decrease pattern. No significant differences in fungal relative abundances were observed across the different tillage years (Table S2). *Blastocladiomycota* was undetected in Y0 but present at very low levels (<1%) in Y5, Y20, and Y60. Eight out of the top 20 fungal families (ranked by relative abundance) showed significant differences (Table S3).

The relative abundances of *Rhizopodaceae*, *Teratosphaeriaceae*, and *Pseudeurotiaceae* in the Y0 plot were significantly lower than those in the plots with longer cultivation durations. Conversely, the relative abundances of *Geoglossaceae*, *Clavicipitaceae*, *Nectriaceae*, and *Trichocomaceae* in the Y0 plot were higher than in the other three plots (Fig. 3D).

### Bacterial and fungal biomarkers under different tillage years

**Figure 4:**
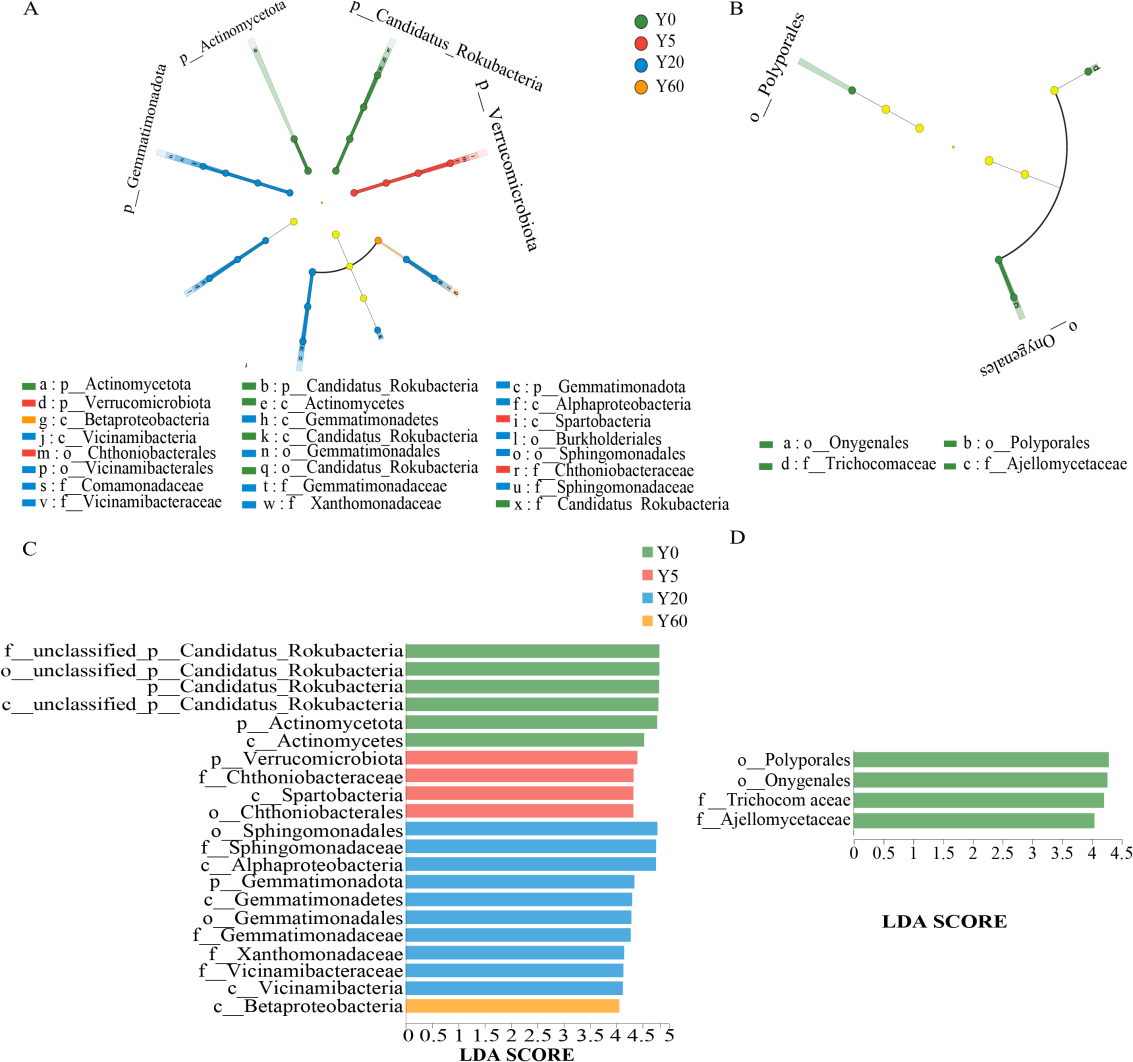
The phylogenetic dendrogram shows the phylogenetic structure (from phylum to family) of soil bacteria (A) and fungi (B) under different cultivation years. Nodes in different colors in the figure indicate microbial taxa that are significantly enriched in the corresponding cultivation years and have a significant impact on intergroup differences; light yellow nodes represent microbial taxa that show no significant differences in different groups or have no significant impact on intergroup differences. Linear discriminant analysis effect size (LEfSe) analysis of differential abundances (LDA>4, *p* < 0.05) of bacteria (C) and fungi (D) in microbial communities under different cultivation years.

LEfSe analysis was conducted to identify microbial taxonomic differences from the phylum to family levels across different tillage durations (LDA > 4, *p* < 0.05). The Y0 group showed six significantly different bacterial taxa, including *Candidatus Rokubacteria* and *Actinomycetota* (including *Actinomycetes*). The Y5 group exhibited significantly enriched *Verrucomicrobiota*, while the Y20 group contained ten distinct bacterial taxa, including *Gemmatimonadota*, *Vicinamibacteria*, *Alphaproteobacteria*, *Burkholderiales*, and *Xanthomonadaceae*. The Y60 group was characterized by *Betaproteobacteria* (Figs. 4A, 4C).

Fungal biomarkers showed fewer significant differences compared to bacteria, with only *Onygenales* (including *Ajellomycetaceae*), *Polyporales*, and *Trichocomaceae* identified in the Y0 group (Figs. 4B, 4D).

### Predictive analysis of microbial community functions

**Figure 5:**
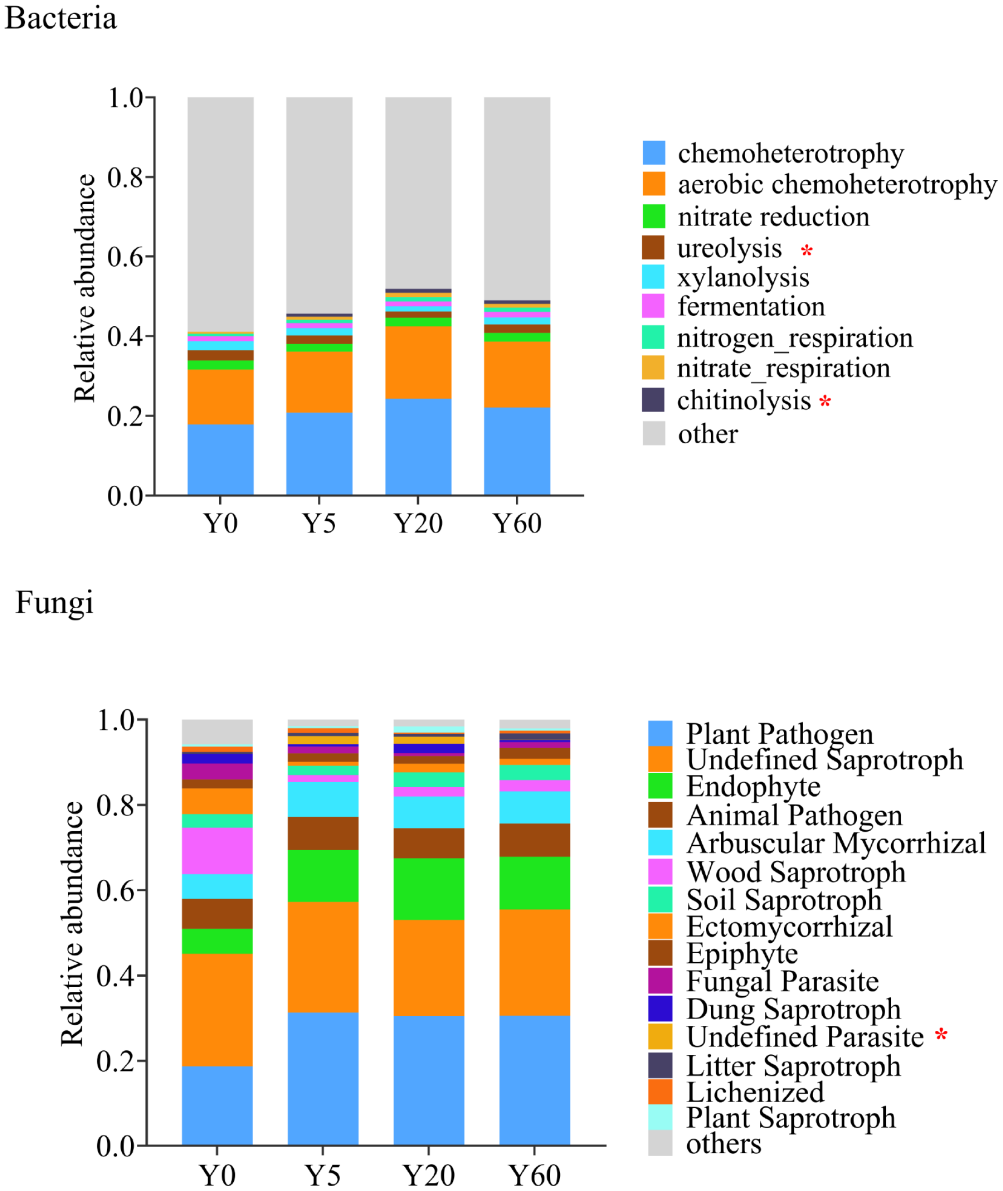
Relative abundances of soil bacterial and fungal functional communities under different tillage years. * indicates *p* < 0.05.

As shown in Fig. 5, bacterial functions were significantly enriched (>1%) in chemoheterotrophy (17.84%–24.27%), aerobic chemoheterotrophy (13.75%–18.15%), nitrate reduction (1.93%–2.3%), ureolysis (1.55%–2.54%), xylanolysis (1.27%–2.3%), fermentation (1.23%–1.74%), nitrogen respiration (0.58%–1.12%), nitrate respiration (0.507%–1.07%), and chitinolysis (0.76%–1.03%). With increasing tillage duration, chemoheterotrophy, aerobic chemoheterotrophy, nitrogen respiration, nitrate respiration, and chitinolysis initially increased before declining. In contrast, xylanolysis and chitinolysis showed significant variations across different tillage durations. For fungal functions, the Plant Pathogen function initially increased with tillage before stabilizing, while Endophyte and Undefined Parasite functions increased over time. Conversely, the Wood Saprotroph, Ectomycorrhizal, and Fungal Parasite functions decreased, with significant differences observed in the Undefined Parasite function.

### Correlation analysis between soil factors and microorganisms

**Figure 6:**
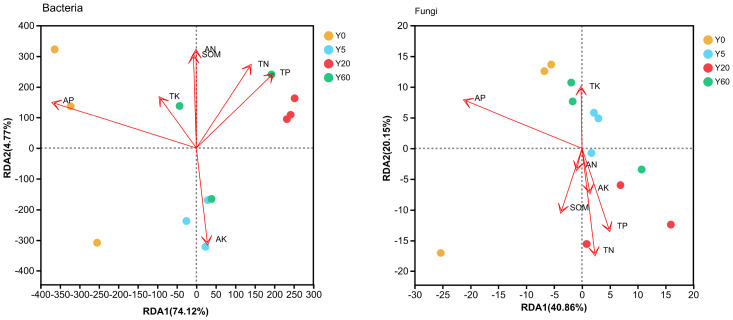
Redundancy analysis (RDA) of microbial communities and soil environmental factors under different tillage years. SOM, soil organic matter; TN, total nitrogen; TP, total phosphorus; TK, total potassium; AN, available nitrogen; AP, available phosphorus; AK, available potassium.

To identify potential environmental drivers, correlations between bacterial and fungal community abundances and soil factors were analyzed. RDA analysis revealed that the first two axes explained 78.89% and 61.01% of the variation in soil bacterial and fungal communities, respectively. TN, TP, AN, and SOM were positively correlated with bacterial community composition, while AP was negatively correlated with bacterial communities. For fungal communities, TK was positively correlated, and AP was negatively correlated (Fig. 6).

**Figure 7:**
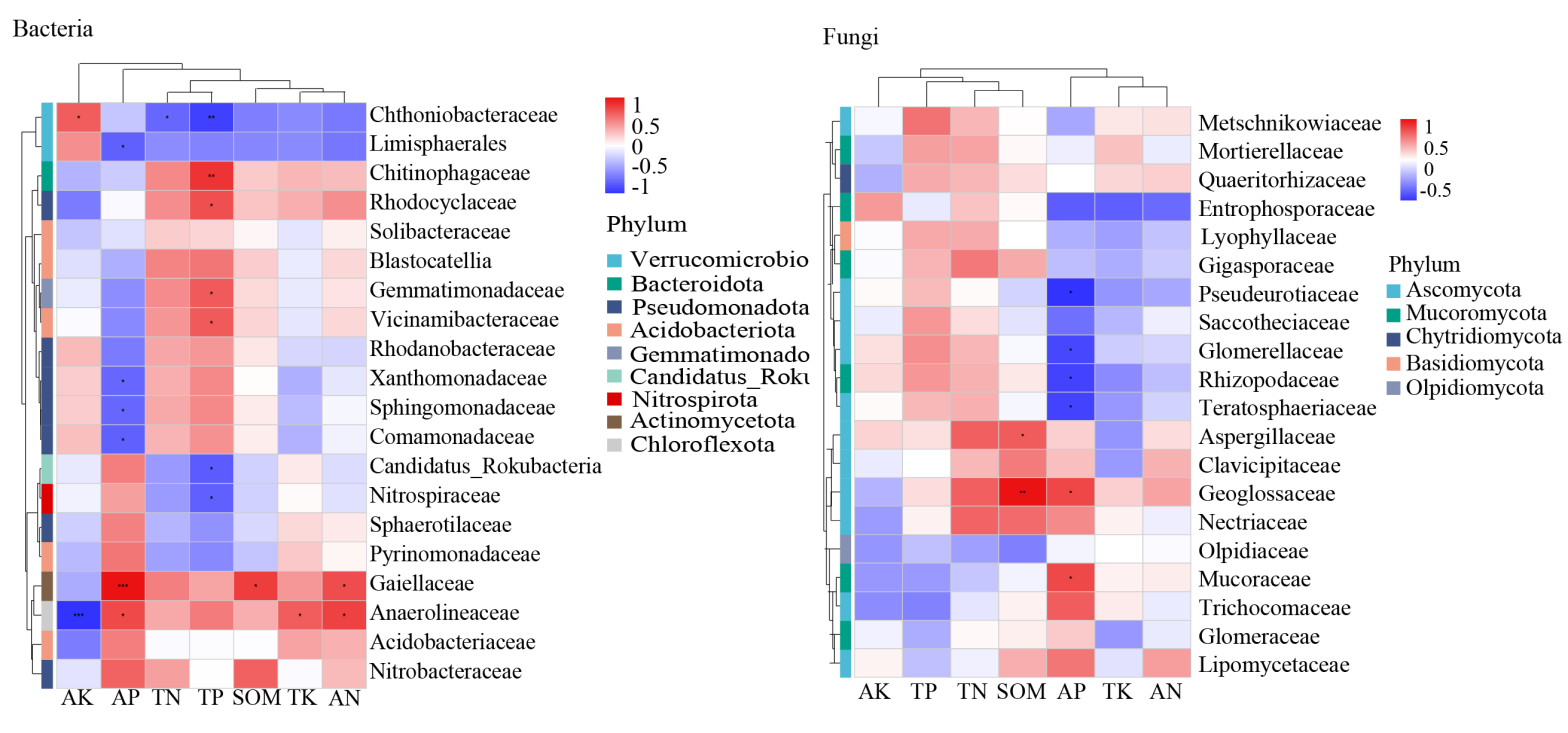
Correlation heatmap between rhizosphere soil physicochemical properties and bacterial and fungal communities across different cultivation durations.

The correlation heatmap (Fig. 7) revealed that TN, TP, AN, and SOM were positively correlated with the relative abundances of *Gaiellaceae*, *Anaerolineaceae*, and *Chitinophagaceae* but negatively correlated with *Chthoniobacteraceae*. Among fungi, AP was positively correlated with Mucoraceae and *Geoglossaceae*, while negatively correlated with *Teratosphaeriaceae*, *Glomerellaceae*, *Rhizopodaceae*, and *Pseudeurotiaceae*.

**Figure 8:**
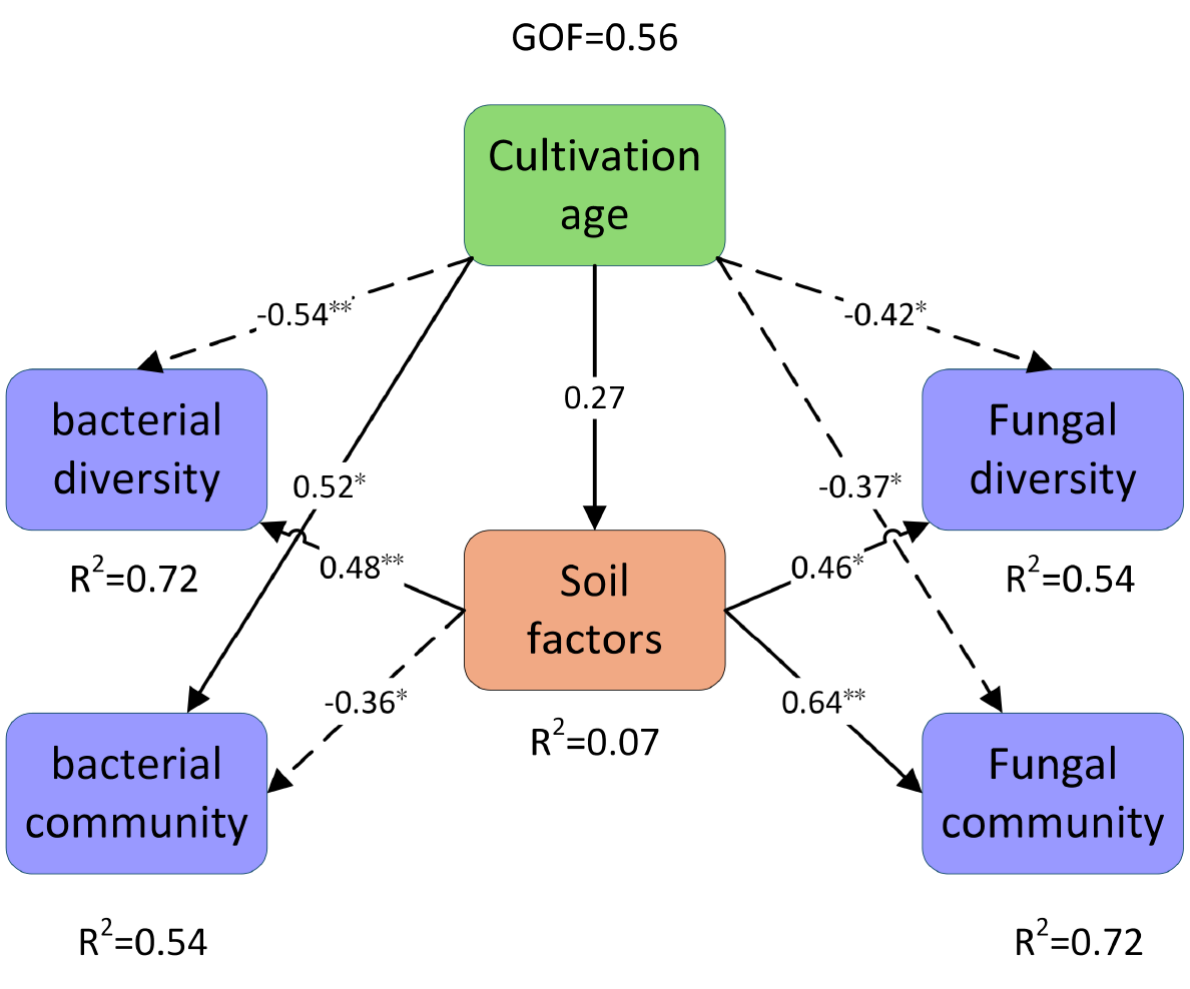
Illustrates the effects of tillage duration, soil factors. (SOM, Soil Organic Matter; TN, Total Soil Nitrogen; TP, Total Soil Phosphorus; TK, Total Soil Potassium; AN, Available Soil Nitrogen; AP, Available Phosphorus; AK, Available Potassium), soil microbial community diversity (Chao index and Shannon index), and soil microbial community structure (NMDS1, NMDS2) using the Partial least-squares analysis. The values above the lines represent path coefficients. Solid lines indicate positive path coefficients, while dashed lines indicate negative path coefficients. Asterisks denote the significance level of the effects: *p < 0.05, **p < 0.01, ***p < 0.001.

Partial least squares analysis indicated that cultivation duration and soil factors had significant effects on microbial communities, with specific path coefficients and significance levels (Fig. 8). Cultivation duration had a significant negative effect on the diversity of soil bacteria (path coefficient = −0.54, *p* < 0.01) and fungi (path coefficient = −0.42, *p* < 0.05), and a significant positive effect on bacterial community composition (path coefficient = 0.52, *p* < 0.05). In contrast, it had a significant negative effect on fungal community composition (path coefficient = −0.37, *p* < 0.05). Soil factors exhibited a significant positive effect on the diversity of both bacteria (path coefficient = 0.48, *p* < 0.01) and fungi (path coefficient = 0.46, *p* < 0.05), a significant negative effect on bacterial community composition (path coefficient = −0.36, *p* < 0.05), and a significant positive effect on fungal community composition (path coefficient = 0.64, *p* < 0.01).

## Discussion

### Changes in rhizospheric microbial community structure under different tillage years

Intensive cultivation and improper land use have led to significant soil degradation and fertility loss, posing a threat to soil quality and the long-term viability of agricultural ecosystems (Gomiero, 2016). Tillage practices induce substantial alterations in soil physicochemical properties, influencing microbial interactions, species distribution, and, consequently, the size and diversity of microbial communities (Altieri, 1999). Changes in the Chao1 index of bacteria were generally negatively correlated with soil nutrient differences across soils with varying cultivation durations. These alterations likely stem from disruptions in soil chemical and physical properties caused by anthropogenic tillage practices (Sun et al., 2023), chemical fertilizer application, and the incorporation of fresh crop residues (Zuber & Villamil, 2016). Such disturbances further destabilize bacterial habitats, leading to fluctuations in microbial communities (McNaughton, 1994).

The Chao1 and Shannon diversity indices for fungi decreased with the lengthening of cultivation duration. Specifically, fungal community richness declined significantly in the early stages of cultivation, while both fungal diversity and richness tended to stabilize and converge over time. A combined analysis of NMDS results and changes in community diversity revealed that bacterial communities were more sensitive to long-term cultivation than fungal communities (Anderson et al., 2017), supporting Anderson’s findings.

With increasing tillage years, soil microbial community composition underwent significant changes. In this study, *Pseudomonadota, Acidobacteriota, Actinomycetota*, and *Verrucomicrobiota* were the dominant bacterial phyla across all tillage years, consistent with previous studies on soils from similar regions (Cai et al., 2021). Different cultivation durations led to variations in the composition ratios of rhizosphere microorganisms. The relative abundance of the phylum *Proteobacteria* first increased and then decreased with the extension of cultivation duration. *Proteobacteria* are typically regarded as symbiotic bacteria, which thrive under nutrient-rich conditions (Ahn et al., 2016; Peiffer et al., 2013). This trend is supported by the higher nutrient contents (AN, TN, TP, SOM) observed in the 20-year cultivation group compared to other groups. However, as cultivation duration increased and soil nutrient status declined, the relative abundance of *Proteobacteria* decreased or stabilized after 20 years of cultivation. The phyla *Actinobacteriota*, *Gemmatimonadota*, and *Bacteroidota* also varied with soil nutrient status, behaving as eutrophic taxa (Ghosh et al., 2016; Zhao et al., 2017). In contrast, *Acidobacteriota* and *Verrucomicrobiota*, which are oligotrophic taxa, exhibited little change in their proportions across farmlands with different cultivation durations. This may be due to their limited ability to absorb, metabolize, or degrade low-nutrient substances in eutrophic environments (Cai et al., 2021). The relative abundance of *Actinobacteriota* decreased significantly at the beginning of cultivation but stabilized with further cultivation. Families such as *Sphingomonadaceae*, *Rhodocyclaceae*, *Rhodanobacteraceae*, *Comamonadaceae*, and *Xanthomonadaceae*, belonging to *Proteobacteria*, followed the same variation trend as the phylum *Proteobacteria*. Similarly, *Chthoniobacteraceae* and *unclassified_o__Limisphaerales*, belonging to *Verrucomicrobiota*, showed consistent variation patterns with *Verrucomicrobiota*; *Solibacteraceae*, *Vicinamibacteraceae*, *Pyrinomonadaceae*, and *Acidobacteriaceae*, members of *Acidobacteriota*, displayed trends in line with *Acidobacteriota*; and *Gemmatimonadaceae*, a family under *Gemmatimonadota*, exhibited a similar variation trend as its phylum. Beneficial microorganisms, including *Sphingomonadaceae*, *Rhodanobacteraceae*, *Comamonadaceae*, *Xanthomonadaceae*, and *Gemmatimonadaceae*, play pivotal roles in antagonizing plant pathogens, improving soil nutrients, and promoting crop growth during potato cultivation (Bak et al., 2025; Asaf et al., 2020). The abundance of these eutrophic microbial taxa increased with the extension of cultivation duration, peaking at Y20, before significantly decreasing. This suggests that long-term cultivation has a negative impact on the beneficial rhizosphere microorganisms in potato soils.

*Ascomycota, Mucoromycota*, and *Basidiomycota* were the dominant fungal groups across different tillage years. The consistently high proportion of *Ascomycota* across all tillage durations aligns with studies of various soil types (Lü et al., 2019), highlighting their role as primary decomposers in agricultural ecosystems (Jiang et al., 2021). *Mucoromycota*, dominant cellulose-decomposing fungi in China’s Alpine-cold regions, such as Tibet and the Greater Khingan Mountains (Wachowska & Rychcik, 2023), are particularly adapted to low-temperature environments (Orchard et al., 2017). Their proportion increased with the duration of tillage. The proportion of *Basidiomycota* fluctuated in response to changes in SOM across different tillage years, likely due to their primary role in decomposing recalcitrant organic matter. As crop litter or residue increases in recalcitrant organic matter over time, the abundance of *Basidiomycota* is expected to shift accordingly (Jiang et al., 2021; Yan et al., 2020a). *Glomerellaceae*, a family within *Ascomycota*, is a highly destructive pathogenic fungus of potatoes, responsible for black scurf on tubers and significant yield losses (Nepal et al., 2025). *Glomeraceae*, a family within *Mucoromycota*, is categorized as arbuscular mycorrhizae and forms a symbiotic relationship with plant roots (Martín-Cacheda et al., 2026). This symbiosis enhances plant nutrient uptake efficiency, improves plant stress resistance (Zhang et al., 2022), and contributes to increased plant disease resistance (Powell et al., 2009). Fungal abundance data across different cultivation durations indicated that pathogenic fungi abundance increased with longer cultivation, while the abundance of beneficial fungal taxa decreased over time. This suggests that long-term cultivation may elevate the risk of potato diseases.

### Changes in rhizospheric microbial taxa and functions under different tillage years

To identify the key microbial taxa in the rhizospheric soil of potatoes under different tillage durations, bacterial and fungal abundances were compared from the phylum to family levels across tillage years using LEfSe software. The results revealed that long-term tillage led to significant differences in the key microbial taxa across the various tillage durations. *Candidatus Rokubacteria* and *Actinomycetota* were notably enriched in soil with zero years of tillage. *Candidatus Rokubacteria* plays a critical role in nitrogen and sulfur cycling (Butterfield et al., 2016; Shi et al., 2021), indicating that these bacteria have adapted to the current soil environment and occupy stable ecological niches. After five years of tillage, *Verrucomicrobiota* was significantly enriched, with its populations involved in nitrogen fixation (Khadem et al., 2010) and polysaccharide degradation (Martinez-Garcia et al., 2012). As tillage extended to 20 years, key microbial taxa were enriched from phyla to lower taxonomic levels. Bacterial taxa, from phyla to families, participated in the nutrient cycling of soil carbon, nitrogen, phosphorus, and organic matter. *Gemmatimonadota* demonstrated strong responsiveness to soil carbon, nitrogen, and phosphorus levels (Zhu et al., 2018), likely acting as the most active bacterial community in soil after 20 years of tillage, significantly increasing with continuous agricultural practices. *Vicinamibacteria* are primarily aerobic chemoorganotrophs that utilize protein substrates and monosaccharides (Dedysh et al., 2022), while *Alphaproteobacteria* are involved in nitrogen fixation, organic matter decomposition, and plant growth promotion (Yarwood, Myrold & Högberg, 2009; Zhang & Xu, 2008). *Burkholderiales* contribute to phosphorus solubilization (Ortega et al., 2024) and nitrification (ammonia oxidation) (Philippot et al., 2013). *Xanthomonadaceae* are hydrocarbon degraders (Lueders et al., 2006), and their abundance is strongly correlated with soil SOC and TN (Li et al., 2017). These taxa reached the highest abundance at the 20-year cultivation duration, with most belonging to the phylum *Proteobacteria*, which was positively correlated with changes in soil nutrients. When tillage extended to 60 years, the abundance of significant taxa decreased substantially. In contrast to bacteria, significant fungal biological groups decreased with increasing tillage years, with key taxa only significantly enriched in the soil with zero years of tillage, predominantly at the order and family levels. Both *Onygenales* and *Trichocomaceae* are saprophytic fungi linked to soil physical properties, such as soil porosity (Bohacz et al., 2022) and aggregates. *Polyporales*, wood-inhabiting fungi, are primarily involved in carbon cycling and alleviating soil carbon limitation (Tedersoo et al., 2014). However, key microbial taxa in both bacteria and fungi decreased with prolonged tillage, and the significant functions performed by microorganisms became more uniform with intensified tillage practices.

Tillage duration not only impacts the changes in key microbial taxa but also drives the ongoing succession of functional groups in soil bacteria and fungi. Microbial functions were analyzed using FAPROTAX and FUNGuild functional prediction software. This study revealed that as the duration of artificial tillage increases, particularly with the application of chemical fertilizers and the incorporation of crop residues, functions related to chemoheterotrophy, aerobic chemoheterotrophy, and ureolysis were significantly enhanced. These functions gained a competitive edge over autotrophic microorganisms. Moreover, functions involved in organic matter decomposition, such as chitinolysis and aromatic compound degradation, also increased with extended tillage. The enhancement of these functions facilitates organic matter breakdown and nutrient release into the soil (Ling et al., 2021; Qin et al., 2015). For fungal functions, the most pronounced differences were observed between the Y0 group and other tillage years. Specifically, the abundance of plant pathogens and endophytic fungi increased with longer tillage durations, while saprotrophic fungi declined—a trend also seen in long-term tea plantation tillage (Wang et al., 2023). It is well-documented that an increase in plant pathogen abundance can lead to diseases or negatively affect host growth (Guo et al., 2019). In contrast, ectomycorrhizal fungi decreased with prolonged tillage. Recent studies suggest that ectomycorrhizal fungi directly degrade and acquire organic nitrogen (Crowther et al., 2019). The decline in ectomycorrhizal abundance under prolonged tillage is likely due to soil disturbance and nutrient mixing, which elevate soil nutrient levels, thereby hindering the formation of stable fungal mantles and restricting ectomycorrhizal proliferation.

### Relationship between soil chemical properties and microbial communities under different tillage years

Long-term conventional tillage can significantly alter soil quality (Laudicina et al., 2015), thereby directly or indirectly affecting the community structure of soil microorganisms (Sa et al., 2018; Zheng et al., 2023). The results of this study indicated that extended tillage significantly impacted soil nutrient contents (Table 1), with soil nutrients being key factors influencing microbial communities (Wang et al., 2022). Partial least squares analysis provided strong evidence for the effects of cultivation duration and soil factors on microbial communities. RDA and the correlation heatmap revealed that TN, TP, SOM, and AN were closely linked to the soil microbial community structure. Eutrophic microbial taxa, such as *Proteobacteria, Actinobacteriota, Bacillota*, and *Bacteroidota*, are better adapted to thrive in soil environments with high organic matter content and nutrient availability. The incorporation of crop residues into the soil further promotes their growth and reproduction (Cui et al., 2022; Ghosh et al., 2016). Beneficial microbial taxa, including *Sphingomonadaceae*, *Rhodanobacteraceae*,*Comamonadaceae*, *Xanthomonadaceae*, and*Gemmatimonadaceae*, are also eutrophic microorganisms, and their growth is facilitated by high levels of SOM and mineral nutrients. In soils supporting crops like cucumber, tomato, and potato (Wang et al., 2022), high organic matter and nutrient contents increase the abundance of beneficial microorganisms, which in turn produce antimicrobial substances that inhibit the growth of fungal pathogens (Raoul des Essarts et al., 2016). However, as soil nutrient conditions decline, the abundance of beneficial microorganisms decreases, leading to the proliferation of fungal pathogens. Notably, significant differences in AP content were observed across soils with varying cultivation durations; AP was positively correlated with beneficial rhizosphere microorganisms in potato and negatively correlated with pathogens. This may be attributed to the phosphorus limitation of soil microorganisms, exacerbated by long-term conventional tillage (Finzi, 2009), which results in shifts in microbial community structure.

## Conclusions

This study investigated the effects of long-term conventional tillage on rhizosphere soil microorganisms in black soils of alpine regions, focusing on how different cultivation durations and soil nutrient levels influence the microbial community of potatoes. The results revealed that long-term tillage significantly affected soil nutrient contents, which in turn altered microbial diversity, functions, and community composition. Bacteria and fungi exhibited distinct responses to prolonged tillage, with bacteria showing greater sensitivity. Specifically, the abundance of beneficial bacterial taxa increased with higher soil nutrient contents. However, as soil nutrients declined due to long-term tillage, beneficial bacterial taxa diminished, while harmful ones proliferated. In contrast, fungal taxa tended to stabilize and converge over time, but the abundance of fungal pathogens rose as beneficial fungi decreased, negatively impacting the health and sustainability of the potato industry. These findings provide valuable insights for the long-term use of black soils in alpine regions. For the sustainable cultivation of potatoes and other crops, farmers should focus on optimizing soil nutrient management. Where necessary, practices such as protective tillage, fallowing, or green manure crop rotation should be employed to maintain soil fertility and promote long-term sustainability.

##  Supplemental Information

10.7717/peerj.21205/supp-1 Supplemental Information 1 Data on community differences

10.7717/peerj.21205/supp-2 Supplemental Information 2 Original soil data

10.7717/peerj.21205/supp-3 Supplemental Information 3 NCBl GEO code

## Additional Information and Declarations

### Data Availability

The following information was supplied regarding data availability: The raw data are available in the Supplemental Files. The raw sequence reads are available at NCBI GEO: PRJNA1305339.
