# Supplementary material for: Changes in rhizosphere microbial community of potato under farmland with different cultivation years in alpine-cold regions
Source: PeerJ. 2026 Jul 6;14:e21205. doi: 10.7717/peerj.21205 (PMC13348483; doi:10.7717/peerj.21205)
Supplement: Supplemental Information 1 [file peerj-14-21205-s001.doc]

Supplementary Information

Table S1. Relative abundances of the dominant bacterial phyla (relative abundance > 1%) in rhizosphere soils with different cultivation years

| Phylum | | | Y0 | Y5 | | | | Y20 | | Y60 | |  |
| --- | --- | --- | --- | --- | --- | --- | --- | --- | --- | --- | --- | --- |
| Pseudomonadota | | | 4.33±0.73 c | | | 7.7±0.5 b | 11.17±1.21 a | | | 10.14±5.22 a | |  |
| Acidobacteriota | | | · 6.7±0.82 b | | | 8.29±0.43 a | 9.13±0.43 a | | | 9.22±0.69 a | |  |
| Actinomycetota | | | · 13.53±5.22 a | | | 6.67±0.24 b | 5.92±0.58 b | | | 7.22±1.7 b | |  |
| Verrucomicrobiota | | | · 4.3±1.54 d | | | 9.73±1.16 a | 8.9±0.28 c | | | 10.4±4.45 b | |  |
| Candidatus_Rokubacteria | | | · 7.69±2.78 b | | | 10.68±0.5 a | 6.87±0.32 b | | | 8.1±1.27 b | |  |
| Gemmatimonadota | | | · 14.47±0.23 a | | | 8.52±1.33 b | 3.35±0.1 c | | | 7±2.04 b | |  |
| Bacteroidota | | | · 3.17±0.41 c | | | 8.78±0.94 b | 11.39±0.44 a | | | 10±2.66 ab | |  |
| Myxococcota | | | · 8.28±0.59 d | | | 7.21±0.23 c | 6.98±0.12 b | | | 10.86±2.77 a | |  |
| Chloroflexota | | | · 4.67±0.51 b | | | 7.02±0.83 ab | 10.09±0.84 a | | | 11.56±6.01 a | |  |
| Cyanobacteriota | | | · 5.56±1.47 b | | | 8.96±2.33 ab | 8.72±0.84 ab | | | 10.09±2.33 a | |  |
| Bacillota | | | · 7.68±1.35 b | | | 8.11±0.38 ab | 8.14±0.28 ab | | | 9.4±0.91 a | |  |
| Planctomycetota | | | · 10.1±1.7 a | | | 7.57±0.41 b | 6.19±0.08 b | | | 9.48±1.28 ab | |  |
| Nitrospirota | | | · 6.57±1.02 c | | | 8.26±0.52 b | 9.58±0.46 a | | | 8.92±0.6 ab | |  |
| Y0, Y5, Y20, and Y60 represent soils tilled for 0,5,20, and 60 years.Values represent mean ± standard deviation based three replicates.Different lowercase letters indicate significant difference in different sampling location, P<0.05.  Table S2.Relative abundances of the dominant fungal phyla (relative abundance > 1%) in rhizosphere soils with different cultivation years   | Phylum | | | | Y0 | | Y5 | | | Y20 | | Y60 |  | | --- | --- | --- | --- | --- | --- | --- | --- | --- | --- | --- | --- | --- | | Ascomycota | | 8.21±3.93 a | | | | | 6.75±1.03 a | 10.84±2.18 a | | 7.54±5.59 a | |  | | Mucoromycota | | · 8.13±7.9 a | | | | | 7.77±3.73 a | 10.3±2.15 a | | 7.13±1.2 a | |  | | Basidiomycota | | · 21.34±31.96 a | | | | | 1.68±0.3 a | 7.4±8.65 a | | 2.91±1.33 a | |  | | Chytridiomycota | | · 6.26±4.52 a | | | | | 8.15±1.55 a | 11.08±7.17 a | | 7.84±5.39 a | |  | | Olpidiomycota | | · 5.26±3.2 a | | | | | 8.95±1.05 a | 8.77±0.8 a | | 10.35±5.27 a | |  | | Zoopagomycota | | · 6.94±2.79 b | | | | | 12.3±2.16 a | 6.26±2.16 b | | 7.83±1.94 b | |  | | Microsporidia | | 0±1.86 a | | | | | 7.58±3.36 a | 16.67±0.93 a | | 9.09±40.33 a | |  | | Blastocladiomycota | | 8.21±3.93 b | | | | | 6.75±1.03 ab | 10.84±2.18 a | | 7.54±5.59 ab | |  | | Y0, Y5, Y20, and Y60 represent soils tilled for 0,5,20, and 60 years.Values represent mean ± standard deviation based three replicates.Different lowercase letters indicate significant difference in different sampling location, P<0.05.  Table S3.Relative abundances of the dominant fungal Family (top 20) in rhizosphere soils with different cultivation years | | | | | | | | | | | |  | |  | Family | | Y0 | | Y5 | | | Y20 | | | Y60 | | |  | Rhizopodaceae | | 5.46±2.95 b | | 8.3±1.27 ab | | | 11.2±1.62 a | | | 8.38±1.74 ab | | |  | Glomerellaceae | | · 0.25±0.14 a | | 7.21±1.94 a | | | 16.54±11.34 a | | | 9.33±14.6 a | | |  | Glomeraceae | | · 15.52±20.3 a | | 3.98±1.5 a | | | 9.12±4.79 a | | | 4.72±2.13 a | | |  | Aspergillaceae | | · 8.91±2.66 a | | 7.61±2.41 a | | | 9.53±0.99 a | | | 7.28±3.25 a | | |  | Lyophyllaceae | | · 17.32±27.1 a | | 3.34±0.19 a | | | 8.23±5.04 a | | | 4.45±2.55 a | | |  | Teratosphaeriaceae | | · 4.08±0.11 b | | 7.51±2.77 b | | | 14.69±4.15 a | | | 7.05±2.22 b | | |  | Entrophosporaceae | | · 6.07±10.32 a | | 9.71±12.09 a | | | 12.85±4.83 a | | | 4.71±7.97 a | | |  | Saccotheciaceae | | · 2.42±0.52 a | | 7.27±2.42 a | | | 11.19±2.93 a | | | 12.45±13.4 a | | |  | Gigasporaceae | | · 5.79±6.94 a | | 6.89±8.05 a | | | 13.17±4.75 a | | | 7.49±4.19 a | | |  | Geoglossaceae | | · 16.67±11.38 a | | 4.55±0.41 b | | | 6.2±1.49 b | | | 5.92±1.95 b | | |  | Mucoraceae | | · 13.27±3.94 a | | 7.72±1.75 b | | | 4.17±1.6 b | | | 8.18±1.87 b | | |  | Mortierellaceae | | · 7.43±6.98 a | | 6.27±3.75 a | | | 7.92±2.48 a | | | 11.72±8.8 a | | |  | Clavicipitaceae | | · 16.58±8.56 a | | 3.98±3.89 b | | | 10.45±11.64 ab | | | 2.32±1.15 b | | |  | Pseudeurotiaceae | | · 1.35±1.06 b | | 9.98±1.63 a | | | 11.84±2.29 a | | | 10.15±1.83 a | | |  | Olpidiaceae | | · 5.26±3.2 a | | 8.95±1.05 a | | | 8.77±0.8 a | | | 10.35±5.27 a | | |  | Quaeritorhizaceae | | · 6.82±5.06 a | | 6.04±3.72 a | | | 9.97±4.34 a | | | 10.5±1.98 a | | |  | Metschnikowiaceae | | · 4.42±3.07 a | | 7.37±3.99 a | | | 11.5±4.93 a | | | 10.03±3.58 a | | |  | Lipomycetaceae | | · 10.73±1.29 a | | 7.34±2.13 a | | | 8.47±3.88 a | | | 6.78±2.24 a | | |  | Nectriaceae | | · 16.55±12.1 a | | 2.64±0.83 b | | | 6.47±3.6 ab | | | 7.67±5.1 ab | | |  | Trichocomaceae | | · 28.87±9.38 a | | 1.79±1.79 b | | | 0.89±0.89 b | | | 1.79±0.89 b | | |  | Y0, Y5, Y20, and Y60 represent soils tilled for 0,5,20, and 60 years.Values represent mean ± standard deviation based three replicates.Different lowercase letters indicate significant difference in different sampling location, P<0.05.  Table S4.Relative abundances of the dominant bacterial Family (top 20) in rhizosphere soils with different cultivation years | | | | | | | | | | | | | | | | | | | | | | | |  |
|  | Family | Y0 | | | Y5 | | | | Y20 | | Y60 | |
|  | Chthoniobacteraceae | 8±3.08 ab | | | 10.83±0.86 a | | | | 6.55±0.34 b | | 7.95±1.37 ab | |
|  | Candidatus_Rokubacteria | · 14.47±0.23 a | | | 8.52±1.33 b | | | | 3.35±0.1 c | | 7±2.04 b | |
|  | Sphingomonadaceae | · 1.64±0.14 c | | | 8.58±0.42 b | | | | 14.48±0.72 a | | 8.63±2.75 b | |
|  | Gemmatimonadaceae | · 3.19±0.45 b | | | 8.79±0.84 a | | | | 11.35±0.47 a | | 10±2.73 a | |
|  | Rhodanobacteraceae | · 1.13±1.17 a | | | 5.29±1 a | | | | 12.19±7.33 a | | 14.74±22.26 a | |
|  | Vicinamibacteraceae | · 4.65±0.1 c | | | 8.51±0.41 b | | | | 11.16±0.72 a | | 9.01±0.96 b | |
|  | Sphaerotilaceae | · 8.89±1.34 a | | | 8.05±0.83 ab | | | | 6.88±0.22 b | | 9.51±0.93 a | |
|  | Blastocatellia | · 5.14±0.71 c | | | 8.49±0.25 b | | | | 10.34±0.26 a | | 9.37±0.74 ab | |
|  | Comamonadaceae | · 3.15±0.08 c | | | 8.9±1.2 b | | | | 11.97±0.59 ab | | 9.32±2.87 b | |
|  | Gaiellaceae | · 10.98±4.55 a | | | 6.19±0.25 a | | | | 7.8±0.96 a | | 8.36±2.79 a | |
|  | Xanthomonadaceae | · 1.76±0.68 b | | | 8.71±0.99 a | | | | 13.12±0.44 a | | 9.74±5.82 a | |
|  | Pyrinomonadaceae | · 10.01±1.83 a | | | 8.19±0.97 a | | | | 5.64±0.29b | | 9.49±1.57 a | |
|  | Nitrosopumilales | · 3.54±1.25 b | | | 8.13±1.01 ab | | | | 9.85±0.48 a | | 11.81±5.87 a | |
|  | Acidobacteriaceae | · 7.6±1.37 b | | | 7.95±0.54 b | | | | 8.05±0.43 b | | 9.74±0.75 a | |
|  | Nitrobacteraceae | · 9.83±4.5 a | | | 7.27±0.62 a | | | | 8.07±0.6 a | | 8.16±2.56 a | |
|  | Chitinophagaceae | · 3.86±0.81 d | | | 6.38±0.85 c | | | | 10.56±1.07 b | | 12.53±8.15 a | |
|  | Rhodocyclaceae | · 5.11±0.69 d | | | 7.18±0.5 c | | | | 9.79±0.48 b | | 11.25±0.76 a | |
|  | Limisphaerales | · 6.52±1.18 c | | | 10.46±2.03 a | | | | 8.01±0.68 b | | 8.34±0.19 ab | |
|  | Anaerolineaceae | · 7.41±0.3 b | | | 6.48±0.2 b | | | | 7.5±0.16 b | | 11.94±4.2 a | |
|  | Solibacteraceae | · 7.03±0.82 b | | | 8.38±0.62 a | | | | 8.95±0.66 a | | 8.97±0.61 a | |
|  | Y0, Y5, Y20, and Y60 represent soils tilled for 0,5,20, and 60 years.Values represent mean ± standard deviation based three replicates.Different lowercase letters indicate significant difference in different sampling location, P<0.05. | | | | | | | | | | | |
