# Supplementary material for: Changes in rhizosphere microbial community of potato under farmland with different cultivation years in alpine-cold regions
Source: PeerJ. 2026 Jul 6;14:e21205. doi: 10.7717/peerj.21205 (PMC13348483; doi:10.7717/peerj.21205)
Supplement: Supplemental Information 3 [file peerj-14-21205-s003.docx]

database name：*NCBI GEO*

*accession numbers：*PRJNA1305339

Changes in Rhizosphere Microbial Community of Potato under Farmland with Different Cultivation Years in Alpine-cold Region, Aug 12 '25;

 Link：https://submit.ncbi.nlm.nih.gov/subs/sra/SUB15536252/overview
